# Supplementary material for: WordCommentsAnalyzer: A windows software tool for qualitative research
Source: F1000Res. 2018 Sep 4;7:536. Originally published 2018 May 3. [Version 2] doi: 10.12688/f1000research.14819.2 (PMC6137411; doi:10.12688/f1000research.14819.2)
Supplement: Supplementary file 3 [file f1000research-7-17614-s0002.tgz › b2a79496-4ada-47c4-9682-a99ce867836d.zip!/Eight Iranian Health Journals/Journal of Health System Research _32.docx]

© Journal of Health System Research . This work is licensed under CC BY-NC 4.0 (https://creativecommons.org/licenses/by-nc/4.0/).

Volume & Issue: دوره 8، شماره 7؛1391

Title: پیشینه حجامت در ایران و نقش آن در سلامت انسان

http://hsr.mui.ac.ir/index.php/jhsr/article/view/995

امروزه در جهان پزشکی و با پیشرفت‌های فراوانی که در عرصه‌های گوناگون آن حاصل شده است، بسیاری از بیماری‌های لاعلاج را درمان کرده‌اند و یا راه‌های پیشگیری از آن را یافته‌اند. از آن‌جا که اساس این پیشرفت‌ها بر بهره‌گیری از داروهای شیمیایی مبتنی است، زیان‌های فراوانی نیز به دنبال آن عارض می‌شود و به همین جهت توجه به شیوه‌های سنتی و بهره‌گیری از دیدگاه‌های گذشتگان -که ضامن سلامت مطمئن‌تر انسان می‌باشد- رواج یافته است. از جمله این روش‌های سنتی، حجامت است که در فرهنگ پزشکی ایران جایگاه ویژه‌ای دارد و هر چند مخالفان و موافقان این شیوه در درستی یا نادرستی آن دلایل فراوان ارایه می‌کنند، بررسی جایگاه آن در نظام سلامت انسان و به عنوان موضوعی که در طول تاریخ پزشکی کشور ما اهمیت فراوانی داشته است، راهگشای دستیابی به عرصه‌های تازه‌تری در استفاده از تجارب گذشتگان و در پی آن روشنگر پژوهش‌های تازه‌تری در تحقیقات نظام سلامت می‌تواند باشد. نویسنده این مقاله کوشش کرده است تا با بررسی منابع و مآخذی که درباره حجامت و انواع و روش‌های آن مطلبی ارایه کرده‌اند، جایگاه و نقش این روش کهن در پژوهش‌های امروزی و تأثیرات مختلف آن بر سلامت انسان را نشان دهد و با مرور آن دیدگاه‌ها، مجالی برای تأمل و بررسی بیشتر دیدگاه‌های صاحب‌نظران تحقیقات نظام سلامت دراین باره فراهم کند.

Title: بررسی عوامل اجتماعی- فرهنگی مؤثر بر سالمند آزاری در شهر اصفهان

http://hsr.mui.ac.ir/index.php/jhsr/article/view/298

مقدمه: سالمندان در جوامع سنتی از قدرت و منزلت زیادی برخوردار بودند، اما شهری و صنعتی شدن جوامع معاصر و تغییر نوع خانواده، پایگاه اجتماعی سالمندان را دگرگون و آن‌ها را دچار مشکلات فراواني کرده ‌است. کاهش اساسی و مستمر مرگ و میر موجب رشد فزاینده جمعیت سالمند شده که این امر بر موقعیت و منزلت سالمندان و سوء رفتار با آن‌ها بی‌تأثیر نبوده است. هدف اصلي تحقيق حاضر، مطالعه تأثير برخي از عوامل اجتماعي و فرهنگي بر پديده سالمند آزاري در شهر اصفهان بود. روش‌ها: تحقیق حاضر به روش پیمایشی و جامعه آماری آن را سالمندان بالای 60 سال که دچار آزار و اذیت عمدی شده و به مرکز پزشکی قانونی استان اصفهان مراجعه کرده‌ بودند، تشکیل دادند که از بین آن‌ها 62 نفر به عنوان نمونه انتخاب شد. برای جمع‌آوری داده‌ها از پرسش‌نامه محقق ساخته و جهت تحلیل داده‌ها از ضريب همبستگي Pearson و ANOVA بهره گرفته شد. یافته‌ها: بین میزان تحصیلات سالمندان و میزان آزار دیدن آن‌ها (05/0 > P، 52/3 = f)، بین این که سالمند با چه ‌کسی زندگی کند و میزان آزار دیدن آن‌ها (05/0 > P، 42/4 = f) و همچنین بین پایگاه اجتماعی- اقتصادی خانواده و میزان آزار دیدن سالمندان (05/0 > P، 592/0 = r) رابطه معنی‌دار وجود داشت، اما بین سبک زندگی سالمندان و میزان آزار دیدن آن‌ها رابطه‌ای دیده نشد (05/0 < P، 106/0 = r). نتیجه‌گیری: سالمند آزاری فیزیکی در شهر اصفهان پایین است و بیشترین نوع آزار به سالمندان از نوع استفاده از اموال بدون رضایت سالمند می‌باشد. البته پیشگیری از شیوع آزارها به سالمندان ضروری به نظر می‌رسد.

Title: هنجاريابي خرده آزمون‌هاي تشخيص باليني- عصب‌شناختي Kaufman روي كودكان سن 7 تا 11 سالگي شهر ايلام

http://hsr.mui.ac.ir/index.php/jhsr/article/view/449

مقدمه: مجموعه آزمون‌هاي تشخيص باليني و عصب‌شناختي Kaufman (ويرايش دوم) يك آزمون فردي است كه براي سنجش توانايي‌هاي شناختي و پردازش كودكان و نوجوانان سنين 3 تا 18 سالگي ساخته شده است. اين آزمون در زمينه ارزيابي روان‌شناختي، باليني، رواني- تربيتي و عصب‌شناختي در همه سطوح پيش دبستاني و دبيرستاني به متخصصان كمك مي‌كند. اين مجموعه يك ابزار باليني مبتني بر نظريه است كه يك سنجش ناوابسته به فرهنگ ارایه مي‌كند كه بسيار ارزشمند است. هدف اين پژوهش هنجاريابي خرده آزمون‌هاي شناختي Kaufman (ويرايش دوم) بود. روش‌ها: روش اين پژوهش توصيفي بود كه براي وضعيت موجود به كار رفت. جامعه آماري كليه كودكان سن 7 تا 11 سال شهر ايلام بود. حجم نمونه با در نظر گرفتن درجه طمينان 95 درصد، حداكثر خطاي 37/0 و انحراف معيار 3 (حداكثر انحراف معيار خرαده آزمون‌ها) كه از يك نمونه مقدماتي برآورد شد، 250 نفر تعيين گردید. این نمونه به صورت تصادفی به روش طبقه‌بندي با تخصيص متناسب سهمي از مدارس ابتدايي شهر ايلام به صورت برابر از بين دختران و پسران گزينش شد. ابزار جمع‌آوري داده‌ها در اين پژوهش خرده آزمون‌هاي تشخيص باليني- عصب‌شناختي Kaufman بود كه براي الگوي Luria ساخته شده بود. براي تحليل داده‌ها از نرم‌افزار SPSS نسخه‌ 19  استفاده گرديد. یافته‌ها: ضرايب α خرده آزمون‌ها، دامنه‌اي از 66/0 تا 76/0 داشت. ضريب α كل آزمون 73/0 بود. نتايج نشان داد كه بين خرده آزمون‌هاي Kaufman و Wechsler  همبستگي معني‌داري وجود دارد (1/0 = P). ميانگين نمرات هوش خرده آزمون‌ها براي كودكان طيف سني 7 تا 11 سال نشان داد كه هوش پديده‌اي است كه با سن افزايش مي‌يابد و رشد هوش از يك روند سني تبعيت مي‌كند. نمرات هنجاري مقياس‌ها مطابق با نمرات خام، رتبه درصدي آزمون‌ها و درصد افرادي كه اين نمرات را كسب كردند، محاسبه گرديد. نتیجه‌گیری: به كمك مجموعه آزمون‌هاي Kaufman ويرايش دوم مي‌توان نيمرخ جامعي از نقاط ضعف و قوت فرد در جنبه‌هاي مختلف توانايي شناختي را ترسيم نمود. نتايج حاصل از آن مي‌تواند در زمينه ارزيابي روان‌شناختي، باليني، رواني- تربيتي و عصب‌شناختي مورد استفاده قرار گيرد.

Title: مقایسه کارایی فرایند فوتولیز و 3O/UV در حذف بنزن از جریان هوا

http://hsr.mui.ac.ir/index.php/jhsr/article/view/476

مقدمه: ترکیب‌های آلی فرار (Volatile organic compounds یا VOCS) یکی از گروه‌های اصلی آلاینده‌های هوا هستند که آثار مخربی بر محیط زیست و سلامت انسان دارند. با توجه به سرطان‌زا بودن برخی از این ترکیب‌ها از جمله بنزن، امروزه چگونگی کنترل آن‌ها موضوعی مهم در پژوهش‌های زیست‌محیطی به حساب می‌آید. هدف از این مطالعه ارزیابی فرایند ترکیبی 3O/UV در حذف بنزن از جریان هوا بود. روش‌ها: مطالعه حاضر از نوع تجربی و در مقیاس آزمایشگاهی انجام شد. در این تحقیق از سیستم آزمایشی شامل پمپ هوا، پمپ تزریق سرنگی، محفظه اختلاط، روتامتر، ژنراتور تولید ازن و راکتور استیل به طول cm 45 و حجم مفید 35/1 لیتر استفاده شد که در داخل آن یک لامپ 15 وات C-UV با طول موج 254 نانومتر بود. غلظت‌های مشخص بنزن به صورت مداوم در زمان ماندهای مختلف در معرض تابش اشعه‌ C-UV، دوزهای مختلف ازن و 3O/UV قرار گرفتند. میزان غلظت بنزن قبل و بعد از در معرض قرارگیری با متغیرهای ذکر شده با هم مقایسه گردید. داده‌ها با استفاده از آمار توصیفی و استنباطی و به کارگیری آزمون Three way ANOVA مورد تجزیه و تحلیل قرار گرفت. یافته‌ها: در این مطالعه غلظت‌های 10، 20، 30، 50 و 80 ppm از بخارات بنزن از درون راکتور عبور داده شد. سپس در معرض تابش C-UV، دوزهای مختلف ازن و UV و ازن توأم قرار گرفت. نتایج نشان داد که UV به تنهایی تا 2 درصد قادر به حذف بنزن از جریان هوا است، ولی ازن قادر است تا حدود 14 درصد بنزن را از هوا حذف نماید. این در حالی است که کاربرد همزمان 3O/UV می‌تواند تا 24 درصد غلظت بنزن را از جریان هوا کاهش دهد. تحلیل آزمون Three way ANOVA نشان داد که بین متغیرهای دوز ازن، اشعه UV و 3O/UV و میزان حذف بنزن از هوا رابطه معنی‌داری وجود دارد (002/0 P <). نتیجه‌گیری: با توجه به تأثیر اندک UV به تنهایی در تجزیه بنزن از جریان هوا می‌توان چنین نتیجه‌گیری کرد که واکنش‌های (AOP یا Advanced oxidation process) که تولید رادیکال‌های فعال می‌کند، می‌تواند برای تجزیه بنزن به کار گرفته شود. یافته‌ها حاکی از آن بود که درصد حذف بنزن در هنگام استفاده توأم از UV و ازن نسبت به کاربرد هر یک به تنهایی کاملاً چشمگیر بود. آزمون تحلیل واریانس نیز نشان داد که کاربرد همزمان UV و 3O دارای اثر هم‌افزایی مثبت است (001/0 P <).

Title: نقش اعتقادات بهداشتی در انجام رفتارهای پیشگیری‌کننده از بیماری قلبی- عروقی در افراد در معرض خطر

http://hsr.mui.ac.ir/index.php/jhsr/article/view/485

مقدمه: در حال حاضر بیماری‌های قلبی- عروقی بیشترین سهم مرگ و میر ناشی از بیماری‌های غیرواگیر زیر 70 سال (39 درصد) را در بر می‌گیرد. در ایران نیز سهم بیماری‌های قلبی- عروقی، 45 درصد می‌باشد. بیماری‌های قلبی- عروقی بار عظیم بهداشتی، اجتماعی و اقتصادی بر جامعه تحمیل می‌کند و زمانی بیمار را گرفتار می‌نماید که از لحاظ اجتماعی و اقتصادی بیشترین بهره‌وری را داراست. در ابتلا به این دسته از بیماری‌ها، عوامل خطر قابل تعدیل شامل سیگار کشیدن، افزایش فشار خون و ... و عوامل خطر غیر قابل تعدیل شامل سن بالا، سابقه خانوادگی و ... دخالت دارند. این مطالعه با هدف نقش اعتقادات بهداشتی در انجام رفتارهای پیشگیری‌کننده در افراد در معرض خطر بیماری قلبی- عروقی اجرا شد. روش‌ها: این مطالعه توصیفی تحلیلی روی 100 نفر از افراد در معرض خطر بیماری قلبی- عروقی 65-30 ساله مراجعه‌کننده به کلینیک خاتم‌الانبیا یزد در سال 1390 انجام شد. ابزار جمع‌آوری داده‌ها پرسش‌نامه طراحی شده بر اساس سازه‌های مدل اعتقاد بهداشتی، سؤال‌های آگاهی، رفتارهای پیشگیری‌کننده از بیماری قلبی- عروقی و متغیرهای دموگرافیک بود. داده‌ها با ا استفاده از نرم‌افزار SPSS نسخه‌ 16  و آزمون‌های Mann-Whitney، Kruskal-Wallis و همبستگی Pearson تجزیه و تحلیل گردید. یافته‌ها: میانگین سنی افراد شرکت‌کننده در مطالعه 6/9 ± 7/43 بود. 56 درصد از افراد مورد مطالعه زن، بیشتر خانه‌دار (43 درصد)، متأهل (93 درصد) و میزان تحصیلات اکثر آن‌ها زیر دیپلم (37 درصد) بود. میانگین نمرات شدت درک‌شده 4/2 ± 23، حساسیت درک‌شده 6/2 ± 15، موانع درک‌شده 4/4 ± 7/20، منافع درک‌شده 45/3 ± 05/27، آگاهی 8/4 ± 7/22 و رفتار پیشگیری‌کننده از بیماری قلبی- عروقی 71/3 ± 11/18 بود. آزمون آماری همبستگی Pearson بین سازه‌های منافع درک‌شده، موانع درک‌شده، خودکارامدی و آگاهی با رفتار پیشگیری‌کننده همبستگی معنی‌دار در سطح 05/0 نشان داد. نتیجه‌گیری: نتایج به دست آمده مبنی بر عدم همبستگی بین حساسیت درک‌شده و شدت درک‌شده با رفتار پیشگیری‌کننده و وجود همبستگی بین منافع درک‌شده، موانع درک‌شده، خودکارامدی و آگاهی با رفتار بود. بنابراین لازم است که برنامه‌های آموزشی مناسب بر اساس مدل اعتقاد بهداشتی و با تأکید بر سازه‌هایی تدوین گردد که تأثیر بیشتری بر رفتارهای پیشگیری‌کننده از بیماری قلبی- عروقی دارند.

Title: بررسي سبک‌های دلبستگی به عنوان یکی از متغیرهای پیش‌بین در وابستگی به مواد مخدر در جوانان پسر شهر ایلام

http://hsr.mui.ac.ir/index.php/jhsr/article/view/550

مقدمه: اعتياد يا سوء‌مصرف مواد موضوعي جدي است كه جوامع انساني را تهديد مي‌كند. متغيرهاي متعددي بر پیدایش، تداوم و درمان اعتياد مؤثر هستند. دسته‌اي از اين متغيرها به عنوان متغيرهاي پيش‌بين در زمينه مصرف مواد بررسي شدند كه از ميان اين متغيرها مي‌توان به سبك‌هاي دلبستگي اشاره كرد. در روان‌شناسي تحول پيوند عاطفي بين نوزاد و مادر را دلبستگي مي‌گويند. تجارب ناايمن در ارتباط با دلبستگي مي‌تواند از عوامل زمينه‌ساز بعدي در جهت سوق به طرف مصرف مواد باشد. در این پژوهش ارتباط سبک‌های دلبستگی با وابستگی به مواد بررسی شد. روش‌ها: افراد مورد مطالعه شامل 120 جوان دانشجو و 81 جوان معتاد بودند. در این پژوهش برای انتخاب نمونه معتاد از روش نمونه‌گیری در دسترس و با مراجعه به مراکز خودمعرف سازمان بهزیستی و برای انتخاب نمونه غیر معتاد از روش نمونه‌گیری خوشه‌ای چند مرحله‌ای و با مراجعه به دانشگاه‌های شهر ایلام استفاده شد. ابزار پژوهش مقیاس سبک‌های دلبستگی بزرگسالان (Revised adult attachment scale یا (RASS Colins و پرسش‌نامه محقق‌ساخته بود. جهت تحليل داده‌ها از نرم‌افزار SPSS نسخه 19  استفاده شد. یافته‌ها: تحلیل رگرسیون یافته‌ها نشان داد که وابستگی به مواد مخدر با سبک‌های دلبستگی (اجتنابی و اضطرابی) رابطه مستقیم، مثبت و معنی‌دار و با سبک دلبستگی ایمن رابطه معکوس، منفی و معنی‌دار داشت و آن را پیش‌بینی کرد (01/0 > P). نتیجه‌گیری: بر اساس نتایج حاصل از این تحقیق می‌توان نتیجه گرفت که نبود تعامل مناسب بین کودک و مادر در دوران کودکی و محرومیت‌های عاطفی، کودکان را به سمت سوء‌مصرف سوق می‌دهد. همچنین والدین غیر پاسخگو و اعتماد کم به آن‌ها، فاصله زیاد بین مادر و فرزند، احساس خصومت و عدم مراقبت لازم در دوران کودکی، فرد را به سوی مصرف مواد هدایت می‌کند.

Title: ارزیابی عملکرد رزین نانو ذرات آهن (لیواتیت FO36) در کاهش سیانید از محیط آبی

http://hsr.mui.ac.ir/index.php/jhsr/article/view/600

مقدمه: سیانید را می‌توان به روش‌های مختلفی از محیط‌های آبی حذف کرد، اما اغلب این روش‌ها هزینه‌بر می‌باشند. در این مطالعه از رزین تبادل کننده لیواتیت FO36 برای حذف سیانید استفاده شد. روش‌ها: در این تحقیق حذف سیانید از محیط مایی توسط رزین تبادل کننده لیواتیت FO36 بررسی گردید و متغیرهای مختلف از جمله اثر میزان ماده جاذب، غلظت سیانید اولیه، زمان تماس و pH در فاز ناپیوسته مورد بررسی قرار گرفتند. یافته‌ها: نتایج تحقیق حاضر که به صورت فرایند ناپیوسته انجام گرفت، نشان داد که pH محلول و همچنین زمان تماس نانو ذرات در شرایط اسیدی در دستیابی به حداکثر میزان حذف سیانید یک عامل مهم می‌باشد؛ به طوری که حداکثر راندمان حذف در 60 دقیقه تماس حاصل گردیده و با افزایش pH از 7 به 11 در زمان تماس 60 دقیقه، راندمان حذف از 74 به 42 درصد کاهش می‌یابد. همچنین نتايج تحقيق نشان داد كه در میزان دوز 3 گرم بر لیتر رزین و زمان تماس 60 دقيقه و در شرایطی که 7= pH و غلظت سیانيد 5/2 ميلي‌گرم بر ليتر باشد، حداکثر راندمان حذف سيانيد 81 درصد به دست می‌آید. لیواتیت FO36 قدرت باند شدن بالایی دارد و اثر تبادل یون سیانید بر روی رزین تبادل کننده کاتیونی از معادله واکنش درجه اول پیروی می‌کند. نتیجه‌گیری: با توجه به یافته‌های این تحقیق، راندمان حذف سيانيد در ابتدا به سرعت و با گذشت زمان به كندي صورت گرفت و راندمان حذف در pH خنثی و اسیدی بیشتر از شرایط قلیایی بود. با این حال، رزین لیواتیت Fo36 می‌تواند به عنوان یک گزینه مناسب با توجه به قابل احیا بودن آن، جهت کاهش میزان سیانید از پساب صنایع و به خصوص صنایع آبکاری که داری مشکل حضور سیانید در پساب می‌باشد، به کار رود.

Title: بررسی عوامل مؤثر بر استفاده از کلاه ایمنی در بین موتورسواران شهر شیراز

http://hsr.mui.ac.ir/index.php/jhsr/article/view/632

مقدمه: ایران از جمله کشورهایی است که بالاترین میزان مرگ و میر ناشی از حوادث را در دنيا دارد. از جمله گروه‌های آسیب‌پذیر در اين حوادث موتورسواران می‌باشند که 42 درصد حوادث جاده‌ای را به خود اختصاص می‌دهند. این مطالعه با هدف بررسی عوامل مؤثر بر استفاده از کلاه ایمنی در بین موتورسواران شهر شیراز انجام شد. روش‌ها: این مطالعه توصيفي مقطعي روي 400 نفر از موتورسواران 15 تا 60 ساله شهر شیراز صورت گرفت. افراد مورد مطالعه به روش نمونه‌گيري تصادفی ساده انتخاب شد و اطلاعات مورد نياز از طريق پرسش‌نامه جمع‌آوری شد. تحليل داده‌ها با استفاده از نرم‌افزار SPSS نسخه‌ 16  انجام شد. مقدار P کمتر از 05/0 به عنوان سطح معنی‌داری در نظر گرفته شد. یافته‌ها: از بين کليه افراد مورد مطالعه فقط 2/11 درصد گزارش کردند که همیشه از کلاه ایمنی استفاده می‌کنند. اغلب افراد (5/62 درصد) عدم راحتی کلاه ايمنی را به عنوان اصلی‌ترين دليل عدم استفاده از آن گزارش کردند. در اين مطالعه ارتباط معنی‌داری بين استفاده از کلاه ايمنی با شغل و همچنين سطح تحصیلات مشاهده شد (05/0 > P). نتیجه‌گیری: نتايج اين مطالعه نشان داد که میزان استفاده از کلاه ایمنی در جامعه مورد بررسی پایین است. بنابراین ضروری است که اقدامات لازم جهت ارتقای ميزان استفاده از کلاه ایمنی از طريق الزام پليس به رعايت و نیز بهبود راحتی آن‌ به عمل آيد.

Title: ارزيابي مقايسه‌اي کیفیت زندگی سالمندان در رابطه با پوکی استخوان در سالمندان ساكن شرق شهر تهران

http://hsr.mui.ac.ir/index.php/jhsr/article/view/637

مقدمه: پوکی استخوان یکی از اختلال‌های متابولیکی شایع دوران سالمندانی است که کیفیت زندگی سالمندان را تحت تأثیر قرار می‌دهد. بنابراین اين تحقيق با هدف بررسی و مقایسه کیفیت زندگی سالمندان مبتلا و غیر مبتلا به پوکی استخوان انجام شد. روش‌ها: اين پژوهش يك مطالعه توصيفي تحليلي و از نوع مقطعی بود كه روي 424 نفر از سالمندان 60 سال و بالاتر عضو خانه‌های سلامت تحت پوشش اداره سلامت شهرداری‌های مناطق شرق شهر تهران انجام گرفت. افراد به صورت تصادفی انتخاب شدند. روش جمع‌آوری اطلاعات، پرسش‌نامه ‌استاندارد کیفیت زندگی 36SF بود که به روش مصاحبه چهره به چهره تکميل گرديد. جهت تجزیه و تحلیل اطلاعات از آزمون‌های ANOVA، t-test و ضریب همبستگی Spearman و Pearson در SPSS  استفاده گردید. یافته‌ها: در مطالعه حاضر 7/63 درصد از شرکت‌کنندگان را زنان و 3/36 درصد را مردان تشکیل می‌دادند. میانگین سنی شرکت‌کنندگان 34/67 با انحراف معیار 16/7 بود. از مجموع کل سالمندان شرکت‌کننده در مطالعه حاضر 145 نفر (معادل 2/34 درصد) ابتلا به پوکی استخوان را گزارش نمودند. ارتباط آماری معنی‌داری بین میانگین نمره کیفیت زندگی و ابتلا به پوکی استخوان مشاهده گردید. نتیجه‌گیری: یافته‌های به دست آمده نشان داد که پوکی استخوان در سالمندان موجب کاهش سطح کیفیت زندگی می‌گردد. بنابراین به نظر می‌رسد که پیشگیری و درمان زود هنگام این بیماری کیفیت زندگی سالمندان را بهبود می‌بخشد.

Title: مقایسه آمار وزارت بهداشت با آمار مرکز آمار ایران پیرامون موارد خودكشي ثبت شده در ايران

http://hsr.mui.ac.ir/index.php/jhsr/article/view/650

مقدمه: سازمان‌های معدودی به انتشار آمار خودکشی در سطح کشور می‌پردازند. از آن جایی که انجام يك مطالعه كشوري مقایسه‌ای روي موارد خودكشي منتشر شده ضرورتي انكارناپذير است، مطالعه حاضر با هدف مقایسه آمارهای خودکشی وزارت بهداشت با آمار خودکشی مرکز آمار ایران در سال 1383 انجام شد. روش‌ها: در این بررسي مقطعي، کليه آمار ارایه شده توسط وزارت بهداشت درمان و آموزش پزشکی به همراه آمار ارایه شده توسط مرکز آمار ایران، به تفکیک استان وارد نرم‌افزار افزار آماری STATA گرديد. در مرحله بعد، تعداد جمعیت هر استان نیز به مجموعه فوق اضافه شد. سپس با استفاده از مدل آماری Poisson ميزان‌هاي خودكشي محاسبه گرديد. یافته‌ها: در مجموع 3170 مورد خودكشي توسط وزارت بهداشت و 2486 مورد خودكشي توسط مرکز آمار ایران ثبت شده بود. در تمامي استان‌ها به جز ايلام، زنجان، قم، مركزي، همدان و يزد تعداد موارد خودكشي ثبت شده توسط وزارت بهداشت بيشتر از آمار گزارش شده توسط مرکز آمار ایران بود. استان ايلام داراي بالاترين ميزان خودكشي بر اساس آمار مرکز آمار ایران بود؛ در حالي كه بر اساس آمار وزارت بهداشت، استان لرستان داري بالاترين ميزان خودكشي بود. نتیجه‌گیری: بالا بودن ميزان‌هاي خودكشي در استان‌هاي غربي كشور با ساير مطالعه‌های موجود در اين زمينه همخواني داشت. همچنين بخشي از عدم همخواني مابين آمار ارایه شده توسط وزارت بهداشت و مرکز آمار ایران را مي‌توان به شيوه جمع‌آوري اطلاعات توسط هر يك از اين ارگان‌ها نسبت داد. تمامي اين نكات نه تنها توجه خاص براي انجام برنامه‌ريزي‌هاي صحيح در جهت پيشگيري از خودكشي را مطالبه مي‌نمايد، بلكه ضرورت تدوین یک نظام جامع اطلاعات برای ثبت دقیق موارد خودکشی در ایران را نیز مطرح مي‌سازد.

Title: بررسی کارایی تصفیه‌خانه فاضلاب شهرک صنعتی آق‌قلا در حذف ترکیبات ازت، فسفر و اکسیژن مورد نیاز شیمیایی (COD) و مقایسه آن با استاندارد خروجی کشور

http://hsr.mui.ac.ir/index.php/jhsr/article/view/655

مقدمه: تصفیه فاضلاب‌ شهرک‌های صنعتی برای رسیدن به استانداردهای مطلوب زیست محیطی و کنترل عملکرد آن‌ها یک ضرورت می‌باشد. بنابراین مطالعه حاضر با هدف، تعیین کارایی واحد تصفیه فاضلاب شهرک صنعتی آق‌قلا (دارای سیستم لجن فعال با هوادهی ممتد) در حذف ترکیبات ازت، فسفر و اندازه‌گیری اکسیژن مورد نیاز شیمیایی (Chemical oxygen demand یا COD) و مقایسه آن با استاندارد خروجی انجام گرفت. روش‌ها: مطالعه توصیفی- مقطعی حاضر در سال 1390 به مدت 6 ماه انجام شد. در طول مدت مطالعه هر هفته دو بار از فاضلاب ورودی، حوض هوادهی و پساب خروجی نمونه‌برداري صورت گرفت. نمونه‌برداری از ورودی تصفیه‌خانه به صورت مرکب و از سایر واحدها به صورت لحظه‌ای انجام شد. به طور کلی 48 بار از فاضلاب و پساب نمونه‌برداری و آزمایش‌ها انجام شد که این کار بر اساس کتاب استاندارد متد صورت گرفت. در نهایت داده‌های مورد مطالعه توسط نرم‌افزار Excel تجزيه و تحليل شدند. یافته‌ها: میانگین غلظت نیتروژن کل، فسفر و COD ورودی به ترتیب در دامنه 23/4 ± 36/7 میلی‌گرم نیتروژن بر لیتر، 4/26 ± 1/68 میلی‌گرم فسفر بر لیتر و 5/183 ± 2/294 میلی‌گرم بر لیتر بود. میانگین غلظت نیتروژن کل، فسفر و COD در پساب خروجی به ترتیب در محدوده 5/0 ± 1/1 میلی‌گرم نیتروژن بر لیتر، 3/8 ± 19 میلی‌گرم فسفر بر لیتر و 8/9 ± 7/14 میلی‌گرم بر لیتر محاسبه شد که با توجه به استاندارد کشور جهت تخلیه پساب‌های خروجی به آب‌های سطحی (به جز فسفر) در محدوده مجاز بود. نتیجه‌گیری: كيفيت پساب خروجي تصفيه‌خانه شهرك صنعتي آق‌قلا به جز فسفر در سایر پارامترها مطابق استانداردهاي زیست محیطی دفع پساب بود و كارايي اين تصفيه‌خانه تحت فرایند سيستم لجن فعال در حذف آلاينده‌هاي ورودي مطلوب بود. اگر چه در مواردي غلظت فسفر در پساب خروجي بیش از حد استاندارد بود که شاید با مديريت و نظارت دقيق، اين نقیصه نیز قابل برطرف شدن باشد.

Title: رابطه بین رفتارهای حمایتی مدیران و توانمندسازی با میل به ماندن در سازمان بهداشت و درمان صنعت نفت اصفهان

http://hsr.mui.ac.ir/index.php/jhsr/article/view/672

مقدمه: مسأله ترک خدمت کارکنان و نیروهای‌ متخصص یکی از معضلات سازمانی است که بر روند حرکت سازمان اختلال ایجاد می‌کند. همچنین این امر برای مدیر سازمان‌ که در پی حفظ، نگهداری، بهبود و بهره‌‌وری نیروی انسانی خود است، به صورت یک مشکل مطرح است. به کارگیری روش‌های صحیح مدیریتی و همچنین ارتقای سطح توانمندی کارکنان، انگیزه آنان را برای ارتباط پایدار و طولانی با سازمان افزایش می‌دهد. هدف این پژوهش، بررسی رابطه بین رفتارهای حمایتی مدیران و توانمندسازی روان‌شناختی با میل به ماندن در سازمان بود. روش‌ها: این پژوهش در سازمان بهداشت و درمان صنعت نفت اصفهان در پاییز سال 1390 انجام شد. از بین کلیه کارکنان سازمان مذکور (354 نفر)، 187 نفر به شیوه نمونه‌گیری در دسترس برای شرکت در پژوهش انتخاب شدند. ابزارهای پژوهش شامل پرسش‌نامه‌های رفتارهای حمایتی مدیران (Rooney و Gottlieb)، توانمندسازی روان‌شناختی (Spreitzer) و میل به ماندن در سازمان (Hunt و همکاران) بود. برای تجزیه و تحلیل داده‌های پژوهش از ضریب همبستگی Pearson و تحلیل رگرسیون چندگانه (به شیوه همزمان) و از نرم‌افزار SPSS نسخه 18  استفاده شد. یافته‌ها: بین رفتارهای حمایتی مدیران و توانمندسازی روان‌شناختی با میل به ماندن در سازمان رابطه همبستگی ساده و چندگانه وجود داشت (01/0 > P)؛ به طوری که رفتارهای حمایتی و توانمندسازی روان‌شناختی توانستند 12 درصد واریانس متغیر میل به ماندن در سازمان را به طور معنی‌داری تبیین کنند (001/0 > P). نتیجه‌گیری: روش‌های مدیریتی به ویژه رفتارهای حمایتی مدیران مکمل ایجاد یک محیط مناسب برای پرورش کارکنان و افزایش انگیزه آنان است. همچنین برداشت کارکنان از سطح توانمندی خود، انگیزه و احساس تعهدشان به سازمان را افزایش می‌دهد.

Title: بررسي سبک‌هاي يادگیري VARK در دانشجویان دانشگاه‌ علوم پزشکي اصفهان

http://hsr.mui.ac.ir/index.php/jhsr/article/view/707

مقدمه: شناسایی و هدایت سبک‌های یادگیری مناسب نقش مهمی در انتخاب روش‌های یاددهی و انتقال مفاهیم و دانش به فراگیران و در نهایت اعتلای سطح آموزش ایفا می‌کند. هدف از مطالعه حاضر تعيين سبک‌های غالب یادگیری در دانشجويان سال اول دانشگاه‌ علوم پزشکي اصفهان بر اساس مدل VARK در سال 1389 مي‌باشد. روش‌ها: در یک مطالعه توصیفی- تحلیلی، 184 نفر از دانشجویان رشته‌های پزشکی، داروسازی، دندانپزشکی، پرستاری و مدیریت خدمات بهداشتی درمانی در دانشگاه‌ علوم پزشکی اصفهان مورد بررسی قرار گرفتند. جمع‌آوری داده‌ها با استفاده از پرسش‌نامه استاندارد (VARK) صورت گرفت. این پرسش‌نامه پس از ترجمه، روایی آن با استفاده از نظر متخصصین و پایایی آن با محاسبه ضریب Cronbach's alpha (86/0 = α) تأیید شد. تجزیه و تحلیل داده‌ها با استفاده از نرم‌افزار VARK که در محیط Excel طراحی شده بود و نرم‌افزار SPSS نسخه 16 انجام شد. یافته‌ها: در مجموع 122 نفر (3/66 درصد) از نمونه‌های مورد بررسی مؤنث و از نظر مقطع تحصیلی بیش از دو سوم آنان (5/68 درصد) در مقطع دکتری حرفه‌ای (رشته‌های پزشکی، دارو‌سازی و دندانپزشکی) و 5/31 درصد در مقطع کارشناسی (رشته مدیریت خدمات بهداشتی درمانی و پرستاری) بودند. 89 نفر از دانشجویان (4/48 درصد) استفاده از سبک یادگیری تک مدله (Single-modal) و 95 نفر نيز (6/51 درصد) استفاده از سبک‌های یادگیری چند مدله (Multi-modal) را ترجیح ‌دادند. بین جنسیت دانشجویان و استفاده از سبک‌های یادگیری تک مدله (009/0 > P) و نیز بین مقطع تحصيلي و سبک‌های یادگیری (05/0 > P) ارتباط معنی‌داری مشاهده شد. نتیجه‌گیری: دانشجویان پسر استفاده از سبک یادگیری جنبشی/حرکتی و دانشجویان دختر استفاده از سبک یادگیری شنیداری را به میزان بیشتری ترجیح ‌دادند. دانستن سبک یادگیری دانشجویان مهارت ارزشمندی در آموزش می‌باشد که به آموزش‌دهنده در حل مشکلات یادگیری دانشجویان کمک شایانی می‌کند. همچنین این امر به دانشجویان کمک می‌کند تا یادگیرنده‌های مؤثرتری باشند.

Title: مقایسه کیفیت خدمات بهداشتی ارایه شده در پایگاه‌های بهداشتی مشارکتی و دولتی شهرستان ري از دیدگاه مراجعه‌کنندگان

http://hsr.mui.ac.ir/index.php/jhsr/article/view/747

مقدمه: امروزه کیفیت خدمات و رضایت مشتری از بحث برانگیزترین جنبه‌های مدیریتی سازمان‌های خدماتی است و مشتری محوری، استراتژی اصلی تمام سازمان‌های جهان به شمار می‌رود. بنابراین تحقيق حاضر با هدف بررسی و مقایسه کیفیت خدمات بهداشتی و درمانی ارایه شده در پایگاه‌های بهداشتی مشارکتی و دولتی شهرستان ري از دیدگاه مراجعه‌کنندگان به این مراکز در سال 1390 انجام شد. روش‌ها: اين پژوهش يك مطالعه توصيفي تحليلي از نوع مقطعی بود كه از بین افراد مراجعه‌كننده به پايگاه‌هاي بهداشتي درماني دولتي و مشاركتي به صورت تصادفی روی 400 نفر انجام شد. روش جمع‌آوری اطلاعات، ابزار پرسش‌نامه‌ SERVQUAL بود که به روش مصاحبه چهره به چهره تکميل گرديد. برای تجزیه و تحلیل اطلاعات از آزمون‌های مقایسه میانگین ناپارامتریک Mann-Whitney، Kruskal-Wallis و Pearson در SPSS  استفاده شد. یافته‌ها: اختلاف آماري معني‌داري بين شكاف میانگین ادراكات و انتظارات كيفيت خدمات در ابعاد ملموس و فيزيكي (001/0 P =)، تضمين خدمات تعهد شده (009/0 P =) و همدلي (007/0 P =) در بين دو پايگاه وجود داشت. شكاف میانگین ادراكات و انتظارات مراجعه‌كنندگان در ابعاد مذکور در پايگاه‌هاي بهداشتي دولتي در مقایسه با پايگاه‌هاي بهداشتي مشاركتي بیشتر بود. نتیجه‌گیری: اين مطالعه نشان داد كه شكاف كيفيت خدمات در پايگاه‌هاي بهداشتی دولتي بيشتر از پايگاه‌هاي بهداشتی مشاركتي است. بهبود كيفيت خدمات ارايه شده در پايگاه‌هاي بهداشتي دولتي با اجرای مداخلاتي در راستاي بهبود محیط فیزیکی، تضمين خدمات تعهد شده، افزايش همدلي کارکنان بهداشتی با مراجعه‌کنندگان و دريافت بازخورد در خصوص مسایل و نتایج درمان از مراجعه‌کنندگان ضروری به نظر می‌رسد.

Title: بررسي اثر بخشي برنامه آموزشي بر بهبود سبک زندگي بيماران مبتلا به ديابت نوع 2

http://hsr.mui.ac.ir/index.php/jhsr/article/view/708

مقدمه: هدف از انجام اين مطالعه، تعيين اثربخشي برنامه آموزشي بر بهبود سبک زندگي بيماران مبتلا به ديابت نوع 2 بود. روش‌ها: پژوهش حاضر يک مطالعه نيمه تجربي بود که 120 نفر از بيماران مبتلا به دیابت مراجعه کننده به بيمارستان‌هاي منتخب دانشگاه علوم پزشکي تهران (دکتر شریعتی، امیر اعلم، بهارلو) از طريق نمونه‌گيري تصادفي انتخاب و در دو گروه مداخله و مقايسه با تخصيص تصادفي قرار گرفتند. ابزار جمع‌آوري اطلاعات، پرسش‌نامه‌اي مشتمل بر 2 بخش شامل اطلاعات دموگرافيک و پرسش‌نامه سبک زندگي شامل سنجش ابعاد آگاهي، نگرش و عملکرد در مورد رعايت رژيم غذايي و انجام فعاليت فيزيکي بود. برنامه آموزشي بر اساس محتواي پرسش‌نامه تنظيم و طي دو جلسه آموزشي به صورت چهره به چهره براي هر بيمار اجرا شد. داده‌ها در طي دو مرحله قبل از انجام مداخله و سه ماه پس از انجام آموزش در دو گروه جمع‌آوري گرديد. جهت تجزيه و تحليل داده‌ها از نرم‌افزار آماري SPSS نسخه 16 و با توجه به توزيع نرمال داده‌ها از آزمون‌هاي آماري پارامتريک استفاده شد. سطح معني‌داري (05/0 > P) در نظر گرفته شد. یافته‌ها: تجزيه و تحليل نتايج به دست آمده نشان داد که برنامه آموزشي تأثير مثبتي بر افزايش ميانگين نمره ابعاد آگاهي، نگرش و عملکرد در هر دو بعد (انجام فعالیت فیزیکی و رعایت رژیم غذایی) در گروه مداخله داشت که اين افزايش از نظر آماري معني‌دار مي‌باشد (001/0> P). نتیجه‌گیری: نتایج اين مطالعه اهميت آموزش ابعاد مختلف سبک زندگي مانند رعايت رژيم غذايي مناسب و انجام فعاليت فيزيکي را در بهبود ابعاد آگاهي، نگرش و عملکرد بيماران که در مجموع منجر به بهبود سبک زندگي بيماران مي‌شود را تأييد مي‌نمايد.

Title: بررسی میزان پراکنش ذرات معلق (5/2PM، 10PM و TSP) در ایستگاه‌های مرکزی اتوبوس شهری همدان و میزان مواجهه با آن‌ها

http://hsr.mui.ac.ir/index.php/jhsr/article/view/996

مقدمه: هدف این مطالعه اندازه‌گیری غلظت ذرات معلق کل، 10PM و 5/2PM در هوای ایستگاه‌های اتوبوس موجود در میدان مرکزی شهر همدان بود. در نهایت نمایه توزیع غلظت ذرات در ایستگاه‌ها تهیه شد و مخاطرات آن‌ها برای سلامتی افردی که روزانه مدتی از وقت روزانه خویش را در این گونه محیط‌ها سپری می‌کند، ارزیابی گردید. روش‌ها: این مطالعه به ‎صورت مقطعی و توصیفی بود. نمونه‎برداری در 6 ایستگاه و در مقاطع مختلف زمانی به صورت سیستماتیک مکانی- زمانی انجام شد. نمونه‌های هوا به ‎وسیله یک سیستم نمونه‎برداری قابل حمل گرفته شد. نمونه‎برداری ذرات معلق Total suspended particles (TSP) و 10PM با دبی 10 لیتر در دقیقه و در ارتفاع یک متری از سطح زمین انجام شد. برای اندازه‌گیری ذرات معلق 5/2PM از سیستم نمونه‎برداری مجهز به سیکلون و دبی L/min 2 استفاده شد. ذرات معلق جمع شده روی فیلتر استر سلولزی مخلوط وزن شد و به صورت غلظت میکروگرم در متر مکعب بیان شد. دما، درصد رطوبت و سرعت وزش باد در موضع نمونه‌برداری اندازه‌گیری و میانگین آن‌ها ثبت شد. براي مقايسه غلظت ذرات معلق در ایستگاه‌های مختلف از روش One way ANOVA و براي تعيين رابطه بين متغيرها از ضریب همبستگي Pearson استفاده شد. یافته‌ها: در طول نمونه‌برداری میانگین غلظت ذرات معلق کل، 10PM و 5/2PM به ترتیب µg/m3 52/1418 ± 94/1220، 56/217 ± 79/524 و 6/193 ± 386 بود. میانگین غلظت ذرات معلق کل، 10PM و 5/2PMبه ترتیب 7/4، 72/7 و 16 برابر مقادیر استاندارد بود. میزان مواجهه به صورت میانگین وزنی زمانی برای این آلاینده‌ها به ترتیب 872/50، 87/21 و 08/16 µg/m3 به دست آمد که از استانداردهای تعیین شده از سوی سازمان‌های معتبر پایین‌تر بود. بین ذرات معلق با سرعت وزش باد و دمای هوا ارتباط معنی‌داری وجود نداشت (05/0 < P)، اما با رطوبت ارتباط معنی‌دار وجود داشت (05/0 ≥ P). همبستگی بین 5/2PM و 10PM با ذرات معلق کل به ترتیب 74/0 = r و 78/0 = r به دست آمد. نتیجه‌گیری: یافته‌های حاصل از این تحقیق بیانگر این است که افرادی که زمان قابل ملاحظه‌ای را در ایستگاه‌های اتوبوس شهری یا مکان‌های نزدیک به آن مستقر هستند، پتانسیل بالایی برای ابتلا به مشکلات بهداشتی دارند. به نظر می‌رسد که عابرین پیاده و افرادی که دارای مشاغل خیابانی هستند، به دلیل زمان تماس بیشتر نسبت به زمان نمونه‎برداری در این مطالعه، با مقادیر بالایی از ذرات معلق مواجهه باشند.

Title: بررسی تأثیر آموزش بیوتروریسم از طریق دو روش سخنرانی و جزوه آموزشی بر آگاهی کادر درمانی در بیمارستان‌های ایرانشهر

http://hsr.mui.ac.ir/index.php/jhsr/article/view/997

مقدمه: يك حمله بيوتروريستي در غياب تأمين تداركات خاص با استفاده از ويروس‌ها از جمله آبله و ... و يا عوامل آلوده‌كننده آب و مواد غذايي، شالوده بهداشت و درمان ملي را به هم می‌ریزد و عده كثيري از بيماران و مردم عادي را به کام مرگ می‌کشد. مهم‌ترین راه مقابله با اقدامات بیوتروریستی در درجه اول تقویت آگاهی تیم پزشکی و بهداشتی جهت تشخیص و عکس‌العمل سریع در مقابله با این حوادث می‌باشد. مطالعه حاضر با هدف تعيين تأثیر آموزش بیوتروریسم از طریق دو روش سخنرانی و جزوه آموزشی بر آگاهی و نگرش کادر درمانی انجام شد. روش‌ها: پژوهش حاضر یک مطالعه نیمه تجربی است. 100 نفر از کادر درمانی جامعه پژوهش را تشكیل دادند که از طریق سرشماری انتخاب شدند. ابزار جمع‌آوری اطلاعات، پرسش‌نامه بود. یک گروه از طریق ارایه جزوه آموزشی و به گروه دیگر از طریق سخنرانی طی دو جلسه دو ساعته، آموزش داده شد. مرحله دوم پژوهش یک ماه بعد انجام شد. جهت تجزیه و تحلیل اطلاعات از نرم‌افزار SPSS  و آمار توصیفی و استنباطی 2c، Mann-Whitney و Wilcoxon استفاده گردید. یافته‌ها: بر اساس نتایج مطالعه، آگاهي کارکنان مراقبتی قبل از آموزش در هر دو گروه سخنرانی و جزوه آموزشی به ترتیب 54 درصد و 70 درصد در سطح پایین قرار داشت که بعد از آموزش در هر دو گروه به ترتیب 64 درصد و 62 درصد در سطح متوسط قرار گرفت. آزمون آماری Wilcoxon اختلاف معنی‌داری بین آگاهی قبل و بعد از آموزش در دو گروه سخنرانی و جزوه آموزشی نشان داد. نتیجه‌گیری: آموزش از دو طریق کارگاه و جزوه آموزشي بر آگاهي کادر درمانی در مورد بيوتروريسم تأثير مثبت داشت، اما میانگین آگاهی در گروه کارگاه آموزشی بیشتر از گروه جزوه آموزشی بود.

Title: مقایسه نمایه توده بدن با نمایه توده چربی بدن در تعیین اضافه وزن در کودکان

http://hsr.mui.ac.ir/index.php/jhsr/article/view/998

مقدمه: تعیین حساسیت و ویژگی نمايه توده بدن (Body mass index یا BMI) بر مبنای استاندارد (Center for disease control) 2000CDC در مقایسه با نمايه توده چربي بدن (Fat mass index یا FMI) به عنوان اضافه وزن واقعی مورد بررسی قرار گرفت.سپس وضعیت اضافه وزن کودکان بر اساس این دو نمایه مقایسه شد. روش‌ها: این مطالعه مورد شاهدی روی دختران دبستانی 10-8 سال شهر اصفهان انجام شد. پس از تعیین گروه‌های مورد (103 نفر) و شاهد (307 نفر)، قد و وزن کودکان اندازه‌گیری شد و BMI با تقسیم وزن به مجذور قد محاسبه گردید. میزان توده چربی بدن نمونه‌ها با استفاده از دستگاه تجزیه و تحلیل‌کننده ترکیب بدن (Body composition assessment یا BCA) اندازه‌گیری شد. FMI با تقسیم توده چربی بدن بر مجذور قد محاسبه شد. مقادیر BMI بیشتر و مساوی صدک 95 استاندارد 2000CDC به عنوان اضافه وزن، مقادیر کمتر از صدک 95 به عنوان فاقد اضافه وزن، مقادیر FMI بیشتر و مساوی صدک 90 مقادیر مرجع به عنوان اضافه وزن واقعی و کمتر از صدک 90 به عنوان فاقد اضافه وزن در نظر گرفته شد. سپس وضعیت اضافه وزن بر اساس این دو معیار مقایسه گردید. یافته‌ها: میانگین و انحراف معیار FMI و BMI در کل کودکان مورد مطالعه به ترتیب 1/2 ± 6 و3 ± 4/19 بود. حساسیت و ویژگی BMI در تعیین اضافه وزن در مقایسه با FMI به ترتیب 3/57 درصد و 4/98 درصد به دست آمد. 6/1 درصد از کودکانی که بر اساس FMI دارای اضافه وزن نبودند، بر اساس BMI دارای اضافه وزن تشخیص داده شدند. 3/57 درصد کودکانی که بر اساس FMI دارای اضافه وزن بودند، بر اساس BMI نیز دارای اضافه وزن تشخیص داده شدند. نتیجه‌گیری: به نظر می‌رسد که FMI توانایی بیشتری نسبت به BMI در تشخیص افراد دارای اضافه وزن داشته باشد. انجام مطالعه‌های بیشتر در این زمینه پیشنهاد می‌شود.

Title: بررسی کارایی گیاه پالایی برای آلاینده‌های نفتی در مقیاس گلخانه در خاک رسی و شور

http://hsr.mui.ac.ir/index.php/jhsr/article/view/999

مقدمه: خاک آلوده به هیدروکربن‌های نفتی یک مسأله زیست محیطی است که سلامت انسان را تحت تأثیر قرار می‌دهد. گیاه پالایی یک روش مقرون به صرفه برای حذف آلاینده‌های نفتی از خاک می‌باشد. در این مطالعه، اثر گیاه و مواد مغذی بر میزان حذف TPH (Total petroleum hydrocarbons) از خاک در طی فرایند گیاه پالایی بررسی گردید. روش‌ها: خاک مورد مطالعه از واحد نمک‌زدایی اهواز 2 جمع‌آوری و با غلظت 5/2 درصد وزنی- وزنی نفت خام آلوده شد. جمعیت میکروبی و TPH خاک گلدان‌های مورد مطالعه در شروع پژوهش و در پایان ماه سوم اندازه‌ گرفته شد. TPH خاک به وسیله دستگاه GC و جمعیت میکروبی با استفاده از روش شمارش بشقابی هتروتروفیک اندازه‌گیری گردید. تحلیل داده‌ها و رسم نمودار نیز به ترتیب با استفاده از نرم‌افزار SPSS نسخه 17 و Excel صورت گرفت. یافته‌ها: میانگین درصد حذف TPH در خاک کشت شده با گیاه (حدود 20 درصد) بیشتر از آن در خاک بدون گیاه (حدود 7 درصد) بود. به علاوه، میانگین جمعیت باکتریایی هتروتروفیک در خاک کشت شده با گیاه (CFU/g 14/7) بیشتر از آن در خاک بدون گیاه (CFU/g 16/6) بود. همچنین بر طبق نتایج، میانگین درصد حذف TPH و میانگین جمعیت میکروبی در خاک دریافت کننده مواد مغذی، بیشتر از آن‌ها در خاک بدون دریافت مواد مغذی بود. نتیجه‌گیری: هر چند میزان بالای رس و شوری خاک مورد مطالعه تأثیر منفی بر کارایی گیاه پالایی داشت، اما نتایج نشان داد که گیاهان بومی حتی در شرایط نامناسب محیطی عمل گیاه پالایی را به خوبی انجام می‌دهند.

Title: بررسی کیفیت آب رودخانه کرخه با استفاده از آنالیز آماری چند متغیره بر پایه همبستگی و تغییرات داده‌های کیفی

http://hsr.mui.ac.ir/index.php/jhsr/article/view/332

مقدمه: گسترش شهرنشینی و فعالیت‌های صنعتی و کشاورزی در حاشیه رودخانه کرخه و به دنبال آن تخلیه پساب آن‌ها به منابع آب باعث آلودگی آب توسط آلاینده‌های فیزیکی، شیمیایی و بیولوژیکی گردیده است. يکي از مسايل مهم در پايش کيفيت سیستم‌های منابع آب، تشخيص پارامترهای آلاینده اصلی و تفکيک پارامترهاي فيزيکي و شيميايي و رابطه بين آن‌ها می‌باشد. اين امر از آن سبب مهم است که پارامترهاي آلاينده فيزيکي به طور معمول ناشي از طبيعت و اقليم حوضه می‌باشد، اما پارامترهاي آلاينده شيميايي از منابع آلاينده انسان ساخت منشأ می‌گیرند. بنابراين اگر همبستگي بين این دو دسته از پارامترهاي آلاينده زياد باشد، مي‌توانند به طور طبیعی از منبع يکساني ناشی شوند. از طرفي يافتن اين ارتباطات با روش‌هاي آزمايشگاهي (مانند روش تفکیک شیمیایی) ممکن است منجر به صرف وقت زیاد و هزينه‌هاي سنگين شود. روش‌ها: در تحقیق حاضر، برای یافتن پارامترهای کیفی اصلی مسؤول آلودگی آب رودخانه کرخه از روش آماری آنالیز فاکتور اصلی (Principal component analysis یا PCA) و برای يافتن ارتباط بين پارامترهاي فيزيکي و شيميايي از روش آماری تحليل همبستگي کانونيک (Canonical correlation analysis یا CCA) استفاده شد. پارامترهای فیزیکی آب رودخانه کرخه شامل: دبی (Q)، کدورت (Turb)، دما (T)، اکسیژن محلول (Dissolved oxygen یا DO)، کل ذرات معلق (Total suspended solids یا TSS) و هدایت الکتریکی (Electerical conductivity یا EC) و پارامترهای شیمیایی آب شامل: pH، اکسیژن‌خواهی بیولوژیکی 5 روزه (Biological oxygen demand یا BOD5)، اکسیژن‌خواهی شیمیایی (Chemical oxygen demand یا COD)، فسفر کل (Total phosphor یا TP) و نیتروژن کل (Total nitrogen یا TN) در نمونه‌های جمع‌آوری شده از 18 ایستگاه در طول رودخانه کرخه بود که در چهار نوبت (در سال 1385) اندازه‌گیری شدند. یافته‌ها: آنالیز PCA نشان داد که تمامی پارامترهای کیفی انتخاب شده برای بررسی کیفیت آب رودخانه کرخه مهم بودند. در آنالیز CCA، ضریب همبستگی برای سه متغیر استاندارد اول، به ترتیب 896/0، 848/0 و 593/0 بودند که نشانگر این بود که Turb، DO و TSS از بین پارامترهای فیزیکی و BOD5، COD، TP و TN از بین پارامترهای شیمیایی، دارای بیشترین امتیاز در کل متغیرهای کانونیک بودند. نتیجه‌گیری: بر اساس نتايج به دست آمده از تحليل همبستگي کانونيک برای داده‌های کیفی مورد بررسی در این تحقیق، مشخص مي‌شود که منشأ هر دو دسته پارامترهاي آلاينده شيميايي و فيزيکي آب رودخانه، انسان ساخت می‌باشد. روش‌های آماری معرفی شده مي‌توانند به عنوان ابزار پشتیبان، تصمیم‌گیری مناسبی جهت بررسي تغييرات کيفي منابع آب در دسترس در مواردي که حجم زيادي از داده‌های کيفي وجود داشته باشد، توسط تصمیم گیرندگان و مدیران آب کشور مورد استفاده قرار گیرد.

Title: بررسي وجود ارتباط آماري بين پارامترهاي میکروبی و انگلی لجن خشك شده تصفيه‌خانه‌هاي فاضلاب استان چهارمحال و بختیاری

http://hsr.mui.ac.ir/index.php/jhsr/article/view/826

مقدمه: یکی از گزینه‌هاي مطرح دفع لجن، استفاده از آن در زمین‌هاي کشاورزي به عنوان کود می‌باشد که ممکن است به دلیل احتمال وجود انواع میکروارگانیزم‌هاي بیماري‌زا، باعث ایجاد طیف وسیعی از بیماري‌های میکروبی و انگلی در انسان و دام شود. هدف از انجام این تحقیق، بررسي وجود ارتباط آماري مناسب بين پارامترهاي مختلف لجن خشك شده تصفيه‌خانه‌هاي فاضلاب استان چهارمحال و بختیاری بود که مي‌توان از اين پارامترها براي پيش‌بيني كيفيت لجن و ويژگي‌هاي استاندارد مربوط به آن استفاده نمود. روش‌ها: در مطالعه توصیفی- تحلیلی حاضر، سه تصفیه‌خانه فاضلاب شهرهای شهرکرد، فارسان و بروجن در استان چهارمحال و بختیاری انتخاب شدند. دلیل انتخاب این تصفیه‌خانه‌ها، مشابهت‌هایی از نظر شرایط محلی و نیز فرایندی بود. با توجه به کوهستانی بودن منطقه و دارا بودن آب و هوای سرد و زمستانی و تابستان‌های همراه با آفتاب داغ و سوزان، دو فصل زمستان و تابستان انتخاب گردید تا وضعیت لجن در شرایط همراه با کمترین و بیشترین میزان تبخیر مورد بررسی قرار گیرد. در این تحقیق کلیفرم مدفوعی، کلیفرم کل و تعداد تخم انگل به عنوان شاخص‌های میکروبی لجن تعیین گردید و ارتباط آماری بین آن‌ها به وسیله ضرايب همبستگي Pearson و نمودار رگرسیون به دست آمد. یافته‌ها: بهترين ارتباط آماري بين مقادير تخم انگل و كليفرم مدفوعي با ضريب همبستگي، 926/0 برقرار شده است و ارتباط آماري بين مقادير تخم انگل و كليفرم کل، برابر با 919/0 بود. نتیجه‌گیری: با توجه به اين‌كه همه ضرايب همبستگي به دست آمده بيشتر از 9/0 بودند، پارامترهاي ذكر شده داراي ارتباط آماري مناسبي با يكديگر مي‌باشند. از آن‌جایی که بهترين ارتباط آماري بين مقادير كليفرم مدفوعي و تخم انگل به دست آمد، تصفیه‌خانه‌هایی با شرایط آب و هوایی و فرایندی مشابه با تصفیه‌خانه‌های تحقیق موجود می‌توانند با استفاده و کمک گرفتن از ارتباطات آماری و نتایج تجزیه و تحلیل SPSS حاصل از این تحقیق، شرایط میکروبی و انگلی لجن‌های خشک شده تصفیه‌خانه‌های خود را پیش‌بینی کنند.

Title: مقایسه مصرف مواد مخدر و علل گرایش به آن در بین دانشجویان دانشگاه علوم پزشکی تهران و دانشگاه تهران

http://hsr.mui.ac.ir/index.php/jhsr/article/view/829

مقدمه: در حال حاضر اعتیاد و مصرف مواد مخدر به عنوان یکی از چالش‌های مهم اجتماعی، بهداشتی و سیاسی در سراسر جهان مطرح می‌باشد. این مطالعه با هدف، مقایسه مصرف مواد و عوامل مؤثر بر آن در دو دانشگاه علوم پزشکی تهران و دانشگاه تهران انجام شد. روش‌ها: مطالعه حاضر به روش مقطعی- توصیفی صورت گرفت. در این مطالعه 489 دانشجو از دانشگاه تهران و 488 دانشجو از دانشگاه علوم پزشکی تهران از طریق نمونه‌گیری تخصیص متناسب و خوشه‌ای انتخاب شدند. ابزار پژوهش، پرسش‌نامه محقق ساخته بود. داده‌های جمع‌آوری شده به وسیله نرم‌افزار آماری SPSS نسخه 18 و با بهره‌گیری از آزمون‌های آماری t، 2c و Regression analysis مورد تجزیه و تحلیل قرار گرفت. در این مطالعه سطح معنی‌داری 05/0 > P در نظر گرفته شد. یافته‌ها: شیوع مصرف مواد مخدر در دانشجویان دانشگاه علوم پزشکی تهران، 33 درصد و در دانشجویان دانشگاه تهران 5/37 درصد بود. سن شروع اولین مصرف بسیاری از مواد در دوران دانشجویی بود. در هر دو دانشگاه بین مصرف مواد و جنسیت، اعتقادات مذهبی، سابقه مصرف مواد در خانواده، دانشکده ارتباط آماری معنی‌دار وجود داشت (001/0 > P). بیشترین روش ذکر شده در بررسی راه‌های اصلی مواجهه با مواد مخدر، دوستان بود. نتیجه‌گیری: با توجه به نقش دانشگاه‌ها به عنوان مرکز تعلیم و تربیت، پیشنهاد می‌شود که برنامه‌های آموزش بهداشت در رابطه با پیشگیری از مصرف مواد مخدر در مدارس انجام شود و در دانشگاه‌ها تداوم یابد. جنسیت و سن شروع مصرف مواد مخدر باید هم‌چنان در برنامه‌های آموزش بهداشت در رابطه با پیشگیری ادامه پیدا کند. طراحی این برنامه‌ها از دوران مدرسه آغاز گردد و در دانشگاه‌ها نیز تداوم یابد.

Title: بررسی نگرش، هنجارهاي انتزاعي و كنترل رفتاري درك شده دانشجویان در زمينه ترك استعمال قلیان

http://hsr.mui.ac.ir/index.php/jhsr/article/view/836

مقدمه: عادت قليان كشيدن در بين جوانان رو به گسترش است و توجه بیشتر به این مسأله ضرورت دارد. هدف مطالعه حاضر، تعيين نگرش، هنجارهاي انتزاعي و كنترل رفتاري ادراك شده دانشجویان پسر در زمينه ترك استعمال قلیان در سال 1391 بود. روش‌ها: مطالعه حاضر از نوع توصيفي- تحليلي و روش کار به این ترتیب بود که پس از تهیه پرسش‌نامه خودساخته و بررسی روایی و پایایی آن، 90 دانشجوی پسر مصرف کننده قلیان که ساکن در خوابگاه‌های دانشگاه علوم پزشکی تهران بودند، به صورت نمونه‌گيري چند مرحله‌ای انتخاب شدند و پرسش‌نامه‌ها را تكميل کردند. داده‌های جمع‌آوری شده توسط نرم‌افزار SPSS و آزمون‌های 2c و Independent t تحليل گردیدند. یافته‌ها: 9/58 درصد دانشجویان برای ترک استعمال قلیان برنامه‌ریزی کرده بودند و 1/41 درصد برای ترک برنامه‌ریزی نداشتند. با این‌که دانشجویان اعتقاد داشتند که ترک قلیان می‌تواند از بسیاری از بیماری‌ها پیشگیری کند؛ اما اعتیادآور نبودن، رفع اضطراب و خستگی و محروم شدن از ارتباط با دوستان قلیانی، مهم‌ترین اعتقادات آنان برای ادامه مصرف قلیان بود. خانواده و سپس جامعه از دانشجویان انتظار داشتند که این عادت را ترک کنند. دانشجویان باور داشتند که موانعی مانند عدم توانایی نه گفتن به تعارف دوستان، ناتوانی در مقابله با وسوسه کشیدن قلیان و سختی صرف نظر کردن از قلیان در محیط‌های تفریحی دوستانه، ترک مصرف قلیان را مشکل می‌کند. نتیجه‌گیری: در طراحی مداخلات جهت ترک قلیان باید بر حذف نگرش‌های مثبت به قلیان و پرورش مهارت‌های نه گفتن، آرام‌سازی و مقابله با وسوسه‌های کشیدن قلیان متمرکز شد.

Title: بررسی و مقایسه درجه توسعه یافتگی بخش بهداشت و خدمات بهداشتی استان‌های ایران در سال 1387

http://hsr.mui.ac.ir/index.php/jhsr/article/view/839

مقدمه: یکی از گام‌های اساسی جهت سیاست‌گذاری و برنامه‌ریزی در بخش بهداشت و خدمات بهداشتی کشور، اطلاع از وضع موجود این خدمات در استان‌ها می‌باشد. هدف این مطالعه نیز تعیین درجه توسعه یافتگی و رتبه‌بندی استان‌های کشور در بخش بهداشت و خدمات بهداشتی به منظور مقایسه و همچنین شناسایی استان‌های توسعه نیافته در این بخش برای تدوین و طراحی برنامه‌ریزی بهتر بود.   روش‌ها: روش جمع‌آوری اطلاعات در تحقیق حاضر، کتابخانه‌ای و اسنادی بود و کلیه آمارها از مرکز آمار ایران و نشریات وابسته به آن جمع‌آوری گردید. جامعه آماری مورد مطالعه را نیز بخش بهداشت و خدمات بهداشتی 30 استان کشور در سال 1387 تشکیل می‌داد. این تحقیق با استفاده از روش‌های تجزیه به مؤلفه‌های اصلی و تاکسونومی عددی و با در نظر گرفتن 17 شاخص بهداشتی و دسترسی به خدمات بهداشتی به رتبه‌بندی استان‌های کشور در این بخش پرداخت.   یافته‌ها: با انجام محاسبات در روش تاکسونومی عددی فاصله همگنی مشخص گردید و بر اساس آن سه استان خراسان شمالی، خراسان جنوبی و هرمزگان خارج از فاصله همگنی قرار گرفتند، بنابراین از جمع استان‌ها حذف شدند و رتبه‌بندی بین 27 استان همگن باقی‌مانده صورت گرفت.   نتیجه‌گیری: با توجه به درجه توسعه یافتگی به دست آمده استان‌ها، مشخص شد که استان‌های ایلام، چهارمحال و بختیاری، اصفهان، یزد، مازندران، سمنان و گیلان نسبت به سایر استان‌های کشور در بخش بهداشت و خدمات بهداشتی وضعیت بسیار مناسبی داشتند و استان‌های سیستان و بلوچستان، قم، آذربایجان غربی، کهکیلویه و بویراحمد، تهران و کرمان وضعیت نامناسبی داشته‌اند. در همین سال استان‌های ایلام و سیستان و بلوچستان به ترتیب توسعه یافته‌ترین و توسعه نیافته‌ترین استان کشور در این بخش بوده‌اند.

Title: بررسی عوامل مؤثر بر کيفيت هوای داخل در يک ساختمان اداری در شهر تهران

http://hsr.mui.ac.ir/index.php/jhsr/article/view/894

مقدمه: در سال هاي اخير شکايات مربوط به کيفيت هواي داخل ساختمان، به علت تجمع آلوده كننده ها در محیط های داخل، افزايش یافته است. این مطالعه با هدف بررسی عوامل موثر بر کيفيت هوای داخل ساختمان در یک ساختمان اداری در شهر تهران انجام شد. روش کار: در این مطالعه توصیفی – تحلیلی برای اندازه گیری عوامل محیطی موثر بر کیفیت هوای داخل ساختمان از دستگاههای قرائت مستقیم و به منظور بررسی نشانه های سندرم بیماری ساختمان از پرسشنامه کیفیت هوای داخل ساختمان استفاده شد. یافته ها: 9 درصد کارکنان مورد بررسي شرايط محيط کار را نامناسب گزارش کردند و بیشترین شکایت آنها به ترتیب مربوط به احساس سنگيني هوا، خستگي و سردرد بود. شاخص های مورد اندازه گیری شامل دی اکسید کربن، منوکسید کربن، ذرات معلق، ترکیبات آلی فرار، ازون، فرمالدئید، دما، رطوبت و صدا پائین تر از حدود توصیه شده بودند. نتیجه گیری: با اینکه اکثر شاخص های کیفیت هوای اندازه گیری شده در مقایسه با مقادیر مرجع از وضعیت قابل قبولی برخوردار بودند، برخی از کارکنان از کیفیت هوای داخل ساختمان مورد بررسی شکایت داشتند. به نظر می رسد عوامل دیگری نظیر استرس شغلی، رضایت شغلی و ... می تواند احساس کارکنان در مورد کیفیت هوای داخل را تحت تأثیر قرار دهد که می بایست مورد بررسی قرار گیرند.

Title: بررسی اثربخشی مدیریت استرس به شیوه شناختی- رفتاری بر استرس، اضطراب و افسردگی زنان باردار

http://hsr.mui.ac.ir/index.php/jhsr/article/view/496

مقدمه: امروزه مسایل زیادی زنان باردار را در معرض اضطراب و ناراحتی قرار می‌دهد. در اثر این امر با وجود پیشرفت‌های عظیم پزشکی تعداد قابل ملاحظه‌ای از پیامدهای منفی بارداری به وقوع می‌پیوند. مناسب‌ترین درمان‌ها به علت محدودیت‌های دوران بارداری، درمان روان‌شناختی می‌باشد. هدف از این مطالعه، بررسی اثربخشی مدیریت استرس به شیوه شناختی- رفتاری بر استرس، اضطراب و افسردگی زنان در دوران بارداری بود. روش‌ها: بدین منظور از بین 80 نفر از زنان باردار که بالاترین نمره استرس، اضطراب و افسردگی را در پرسش‌نامه Depression anxiety stress scale (42-DASS) کسب کردند، 24 نفر انتخاب شدند. این افراد به طور تصادفي در دو گروه آزمایش (12 نفر) و شاهد (12 نفر) قرار گرفتند. این طرح درمانی طی هشت جلسه هفتگی بر اساس راهنمای درمانی آنتون و همکاران در مرکز بهداشت درمانی شهید رجایی شهر شهرضا برای گروه آزمایش اجرا شد. پرسش‌نامه 42DASS- (خرده مقیاس‌های استرس، اضطراب و افسردگی) در دو مرحله پیش آزمون و پس آزمون تکمیل شد. داده‌هاي جمع‌آوري شده توسط نرم‌افزار SPSS نسخه‌ 16 مورد تجزيه و تحليل قرار گرفت. یافته‌ها: میانگین نمرات وضعیت روانی (استرس، اضطراب و افسردگی) گروه آزمایش در مرحله پس آزمون به طور معنی‌داری کمتر از میانگین نمرات استرس در گروه شاهد بود (001/0 > P). نتیجه‌گیری: با توجه به نتایج این پژوهش، درمان شناختی- رفتاری مدیریت استرس می‌تواند به عنوان شیوه درمانی انتخابی در جهت کاهش استرس، اضطراب و افسردگی دوران بارداری به کار گرفته شود. با این حال برای افزایش میزان اثربخشی مداخله باید علاوه بر توجه به افکار ناکارامد، توجه ویژه‌ای به باورهای هسته‌ای و فرض‌های زیربنایی افراد شود.

Title: بررسي ارتباط بين غلظت ذرات معلق در هواي خارج و هوای داخل بيمارستان حافظ شيراز

http://hsr.mui.ac.ir/index.php/jhsr/article/view/721

مقدمه: در سال‌های اخیر غلظت ذرات معلق در هوا افزایش یافته است. این ذرات می‌توانند اثرات نامطلوبی بر سلامتی انسان بگذارند. مطالعه‌ها نشان دادند که کیفیت هوای داخل ساختمان متأثر از هوای بیرون است. از آن جایی که افراد قسمت قابل توجهی از وقت خود را در فضاهای بسته سپری می‌کنند و اندک مطالعه‌هایی در این زمینه موجود می‌باشد، هدف از این مطالعه اندازه‌گیری غلظت ذرات معلق در هوای خارج و هوای داخل بیمارستان حافظ و تعیین ارتباط بین آن‌ها بود. روش‌ها: مطالعه مقطعی در بيمارستان آموزشي حافظ شيراز از مهر تا آذرماه 1390 انجام شد. نمونه‌برداری به صورت هفته‌اي دو بار از هوای داخل هر بخش بیمارستان و هوای خارج صورت گرفت. تعداد كل نمونه‌هايي كه براي آناليز جمع‌آوري شد، 144 نمونه بود كه شامل 24 نمونه براي هر بخش و هوای خارج بود. نمونه‌برداري با استفاده از دستگاه Aerosol mass monitor مدل 331GT- ساخت SIBATA ژاپن برای اندازه‌گیری غلظت ذرات micron 10Particle matter less than  (10PM) و micron 5/2 Particle matter less than (5/2PM) انجام شد. براي مقايسه غلظت ذرات معلق در بخش‌هاي مختلف بيمارستان با ذرات معلق هواي خارج اطراف بيمارستان از آزمون آماري آناليز One way ANOVA استفاده شد. سپس داده‌ها وارد نرم‌افزار SPSS نسخه 5/11 (version 11.5, SPSS Inc., Chicago, IL) شد و مورد تجزیه و تحلیل آماری قرار گرفت. سپس نتایج به دست آمده با غلظت 24 ساعته ذرات معلق رهنمودهاي Environmental protection agency (EPA) و Word health organization (WHO) مقايسه شد. یافته‌ها: وضعیت کیفی هوای داخل بخش‌های بیمارستان و خارج بیمارستان از لحاظ شاخص کیفیت هوا برای 5/2PM خوب، برای 10PM هوای داخل به میزان 2/95 درصد خوب و هوای خارج در 75 درصد شرایط ناسالم برای گروه‌های حساس را نشان داد. با استفاده از روش Pearson Correlation رابطه معنی‌داری برای 10PM و 5/2PM در هوای خارج و داخل تعدادی از بخش‌ها دیده شد (05/0 < P). نتیجه‌گیری: میانگین نسبت 10PM و 5/2PM هوای داخل بخش‌های بیمارستان به هوای خارج (I/O) به ترتیب 41/1 و 61/0 بود. ارتباط معنی‌داری که بین غلظت ذرات معلق در هوای خارج و داخل بیمارستان وجود داشت، نشان‌دهنده نفوذ این ذرات از هوای خارج به داخل بیمارستان بود. از این رو برای بیمارستان‌هایی که در مناطق پر تردد قرار گرفتند، بهره‌گیری از سیستم‌های مناسب تهویه در کاهش میزان آلودگی هوای داخل بیمارستان مؤثر می‌باشد.

Title: نحوه گذراندن اوقات فراغت اعضای هیأت علمی دانشگاه علوم پزشکی اصفهان با تأکید بر نقش ورزش و تفریحات سالم

http://hsr.mui.ac.ir/index.php/jhsr/article/view/730

مقدمه: از جمله مفاهیم نوینی که معنی واقعی خود را با پیدایش انواع تکنولوژی و صنعتی شدن جوامع در زندگی مردم پیدا نموده است، اوقات فراغت و نحوه گذران آن است. ورزش‌های تفریحی با فعالیت‌های مرتبط با اوقات فراغت از مناسب‌ترین و ضروری‌ترین گونه‏های گذران اوقات فراغت در عصر حاضر است. از این رو این پژوهش به بررسی نحوه گذران اوقات فراغت اعضای هیأت علمی دانشگاه علوم پزشکی اصفهان با تأکید بر فعالیت‌های بدنی پرداخت. روش‌ها: روش تحقیق از نوع توصیفی بود. جامعه آماری را کلیه اعضای هیأت علمی دانشگاه علوم پزشکی اصفهان که 670 نفر بودند، تشکیل دادند. حجم نمونه با یک مطالعه مقدماتی و با استفاده از فرمول Cohen 150 نفر در نظر گرفته شد که به روش طبقه‌ای تصادفی با توجه به متغیر جنسیت و دانشکده انتخاب گردید. ابزار جمع‌آوری اطلاعات پرسش‌نامه اوقات فراغت محقق ساخته بود که پایایی آن با استفاده از روش ضریب Cronbach's alpha 82/0 به دست آمد. یافته‌ها: فعالیت‌های پژوهشی با میانگین 37/3 اولویت اول، امور ورزشی با میانگین 49/2 اولویت دوم، امور فرهنگی و اجتماعی با میانگین 25/2 اولویت سوم و امور شخصی و خانوادگی با میانگین 89/1 اولویت چهارم افراد برای گذراندن اوقات فراغت در سطح 05/0 ≥ α به دست آمد. حفظ تندرستی با میانگین 94/3 مهم‌ترین‌ترین انگیزه پرداختن به فعالیت‏های بدنی در اوقات فراغت بود. همچنین تفاوت معنی‌داری بین فعالیت‏های فراغتی بر اساس جنسیت و سابقه خدمت وجود داشت. نتیجه‌گیری: میزان اوقات فراغت اعضای هیأت علمی در سطح پایینی قرار داشت (44/3 x =). به نظر می‏رسد که باید جامعه را از خصوصیات و شیوه‌های انجام ورزش‌های فراغتی و ضرورت ایجاد شرایط و امکانات مناسب با توجه به برنامه‌ریزی جدی و کار فرهنگی آگاه کرد. همچنین اقشار مختلف جامعه به خصوص قشر دانشگاهی را از تأثیرات مثبت اوقات فراغت و تفریحات سالم آگاه نمود و آن‌ها را هر چه بیشتر به سوی این گونه فعالیت‌ها سوق داد.

Title: ابعاد استرس شغلی و ارتباط آن با اختلالات اسکلتی- عضلانی در پرستاران

http://hsr.mui.ac.ir/index.php/jhsr/article/view/732

مقدمه: اطلاعات ناچیزی در مورد ابعاد استرس شغلی و ارتباط آن با اختلالات اسکلتی- عضلانی Musculoskeletal disorders (MSDs) در پرستاران ایرانی در دسترس است. این پژوهش با هدف بررسی ابعاد استرس شغلی و ارتباط آن با شیوع علایم MSDs در پرستاران شاغل در بیمارستان‎های وابسته به دانشگاه علوم پزشکی شیراز انجام شد. روش‌ها: در این مطالعه مقطعی 385 نفر از پرستاران بیمارستان‎های دانشگاه علوم پزشکی شیراز شرکت نمودند. این افراد به روش نمونه‎گیری تصادفی سیستماتیک انتخاب شدند. نسخه‎ فارسی پرسش‌نامه محتوای شغلی و پرسش‌نامه Nordic برای جمع‎آوری داده‎ها استفاده شد. از آمار توصیفی و آزمون آماری Mann-Whitney U با استفاده از نرم‏افزار SPSS نسخه 5/11 (version 11.5, SPSS Inc., Chicago, IL) برای تحلیل داده‏ها استفاده شد. یافته‌ها: میانگین (انحراف استاندارد) ابعاد آزادی تصمیم‏گیری (50/6) 15/58، نیازهای روان‌شناختی (14/5) 19/38، حمایت اجتماعی (67/3) 67/22، نیازهای فیزیکی (58/2) 03/16 و عدم امنیت شغلی (85/3) 74/7 به دست آمد. بر این اساس ابعاد آزادی تصمیم‏گیری و حمایت اجتماعی در سطح پایین و ابعاد نیازهای روان‌شناختی، نیازهای فیزیکی و عدم امنیت شغلی در سطح بالا قرار داشتند. طی 12 ماه گذشته 9/89 درصد از پرستاران، علایم این اختلالات را در یک یا چند ناحیه از دستگاه اسکلتی- عضلانی بدن خود تجربه کرده بودند که در بین آن‌ها کمردرد شایع‏ترین ناراحتی گزارش شده بود (8/61 درصد). از میان ابعاد استرس شغلی فقط زیر مقیاس بار فیزیکی ایزومتریک با شیوع کلی MSDs ارتباط آماری معنی‌داری داشت. بعد نیازهای روان‌شناختی و نیازهای فیزیکی شغل با اختلالات ناحیه ستون فقرات، بعد نیازهای فیزیکی شغل با اختلالات ناحیه اندام فوقانی و بعد آزادی تصمیم‏گیری، نیازهای روان‌شناختی و نیازهای فیزیکی شغل با اختلالات ناحیه اندام تحتانی از لحاظ آماری ارتباط معنی‌داری داشتند. نتیجه‎گیری: شیوع MSDs در پرستاران مورد مطالعه بالاست. در اغلب پرستاران مورد مطالعه سطح استرس شغلی بالا به دست آمد. بر اساس نتایج به دست آمده، هر گونه برنامه مداخله‏ای جهت پیشگیری از MSDs در پرستاران می‏بایست به ترتیب بر کاهش نیازهای فیزیکی، کاهش فشارهای روان‌شناختی و افزایش آزادی تصمیم‏گیری متمرکز شود.

Title: بررسي تأثير برنامه آموزشی مبتنی بر الگوي توانمندسازي بر میزان HbA1C نوجوانان مبتلا به دیابت نوع یک

http://hsr.mui.ac.ir/index.php/jhsr/article/view/812

مقدمه: هدف نهایی کنترل بیماری دیابت بهبود شاخص HbA1c می‌باشد. با توجه به اهمیت موضوع هدف از انجام مطالعه حاضر تعیین اثربخشی الگوی توانمندسازی بر میزان HbA1C نوجوانان دیابتی نوع یک بود. روش‌ها: پژوهش حاضر یک مطالعه نیمه تجربی بود که در بین نوجوانان دیابتی نوع یک مراجعه‌کننده به مراکز بهداشتی شهر زنجان انجام پذیرفت. 47 نوجوان واجد شرایط به طور تصادفی به دو گروه مداخه و شاهد تقسیم شدند و به مدت 5/2 ماه مورد پیگیری قرار گرفتند. شرکت‌کنندگان ابزارهای پژوهش شامل اطلاعات دموگرافیکی، دانش، خودکارامدی و عزت نفس را تکمیل نمودند. همچنین میزان HbA1C آنان مورد سنجش قرار گرفت. مداخله آموزشی بر اساس گام‌های سه‌گانه الگوی توانمندسازی (درک تهدید، مشکل گشایی و ارزشیابی) اجرا شد. تجزیه و تحلیل داده‌ها به وسیله نرم‌افزار آماری SPSS ویرایش 5/11  صورت گرفت. یافته‌ها: بعد از اجرای برنامه آموزشی اختلاف معني‌داري براي افزایش ميانگين نمره دانش، خودکارامدی و عزت نفس و همچنین کاهش میزان هموگلوبین A1C بين نوجوانان در گروه‌هاي مداخله و شاهد وجود داشت (05/0 > P). نتیجه‌گیری: به نظر می‌رسد که اجراي الگوي توانمندسازي بر ارتقای شاخص بالینی کنترل دیابت مؤثر است. از این رو پیشنهاد می‌شود که این الگو برای سایر رده‌های سنی در سطح وسیع‌تر و نیز برای سایر بیماری‌های مزمن به کار گرفته شود.
